# S51 Family Peptidases Provide Resistance to Peptidyl-Nucleotide Antibiotic McC

**DOI:** 10.1128/mbio.00805-22

**Published:** 2022-04-25

**Authors:** Eldar Yagmurov, Konstantin Gilep, Marina Serebryakova, Yuri I. Wolf, Svetlana Dubiley, Konstantin Severinov

**Affiliations:** a Center Molecular and Cellular Biology, Skolkovo Institute of Science and Technology, Moscow, Russia; b Institute of Gene Biology, Russian Academy of Science, Moscow, Russia; c A.N. Belozersky Institute of Physico-Chemical Biology, Lomonosov Moscow State University, Moscow, Russia; d National Center for Biotechnology Information, National Library of Medicine, Bethesda, Maryland, USA; e Waksman Institute for Microbiology, Piscataway, New Jersey, USA; Pennsylvania State University

**Keywords:** RiPPs, antibiotics, microcin C, S1 family peptidases, peptide-nucleotides

## Abstract

Microcin C (McC)-like compounds are natural Trojan horse peptide-nucleotide antibiotics produced by diverse bacteria. The ribosomally synthesized peptide parts of these antibiotics are responsible for their facilitated transport into susceptible cells. Once inside the cell, the peptide part is degraded, releasing the toxic payload, an isoaspartyl-nucleotide that inhibits aspartyl-tRNA synthetase, an enzyme essential for protein synthesis. Bacteria that produce microcin C-like compounds have evolved multiple ways to avoid self-intoxication. Here, we describe a new strategy through the action of S51 family peptidases, which we name MccG. MccG cleaves the toxic isoaspartyl-nucleotide, rendering it inactive. While some MccG homologs are encoded by gene clusters responsible for biosynthesis of McC-like compounds, most are encoded by standalone genes whose products may provide a basal level of resistance to peptide-nucleotide antibiotics in phylogenetically distant bacteria.

## INTRODUCTION

**FIG 1:**
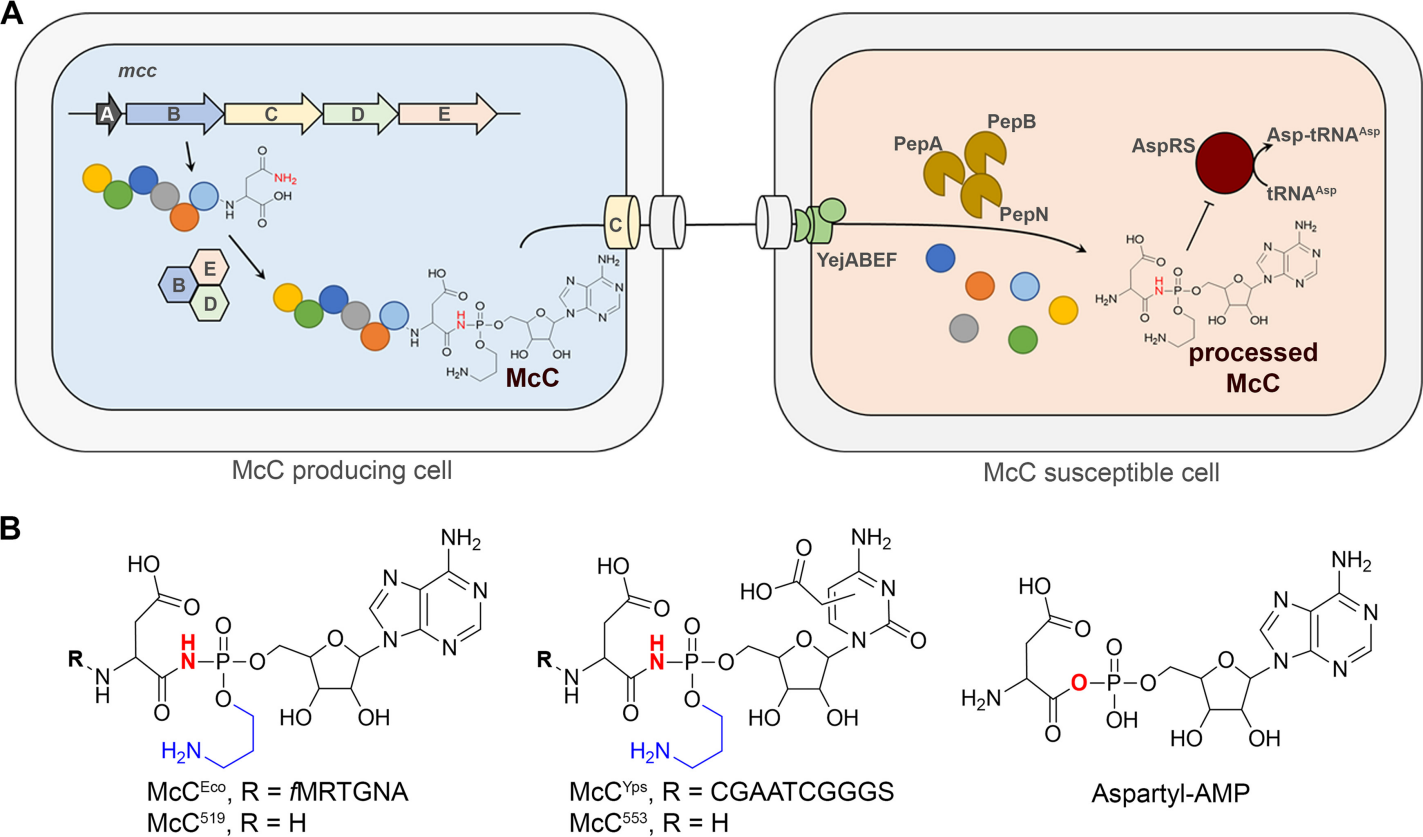
Microcin C (McC) biosynthesis, mode of action, and structural diversity of McC-like compounds. (A) McC^Eco^ biosynthesis by the producing cell containing the *mcc* operon, its uptake by a susceptible cell, and the inhibitory action of the compound on AspRS are schematically shown. The *mccA* gene codes for a precursor peptide, *mccB* encodes a nucleotidyltransferase, *mccC* (an export MFS transporter), and *mccD* and *mccE*, which are required for aminopropyl group installation and self-immunity (*mccE*). (B) Structure of McC^Eco^, a peptidyl-adenylate, and peptidyl-cytidylate McC^Yps^ from Yersinia pseudotuberculosis. The nitrogen atom participating in the formation of nonhydrolyzable phosphoramide linkage is shown in red; the aminopropyl modification is shown in blue. The position of the carboxymethyl group on the cytidyl moiety of McC^Yps^ is currently unknown. The intermediate of the AspRS-catalyzed reaction of tRNA^Asp^ charging (aspartyl-AMP) is shown on the right. McC^519^ and McC^553^ correspond to processed forms of McC^Eco^ and McC^Yps^, respectively.

The Escherichia coli peptidyl-nucleotide antibiotic microcin C (McC) is a prototypical compound of a distinct class of ribosomally synthesized and posttranslationally modified peptides (RiPP) (1). Biosynthesis of McC-like compounds is encoded in *mcc* biosynthetic gene clusters (BGCs) found in the genomes of numerous Gram-negative and Gram-positive bacteria (2, 3). The minimal set of genes required for production of an McC-like compound comprises *mccA*, which encodes a precursor peptide; *mccB*, coding for the ThiF-like nucleotidyltransferase; and a gene whose product is responsible for antibiotic export. Other genes frequently present in *mcc* BGCs are responsible for either additional decorations on the nucleotide part of the final product or for self-immunity of the producer. Upon translation, the MccA peptide is modified by MccB with the formation of peptidyl-nucleotide, in which the C-terminal asparagine is converted to isoasparagine and linked to a nucleoside monophosphate through a nonhydrolyzable phosphoramide linkage (4) (Fig. 1A). The reaction consumes two molecules of triphosphates per one molecule of McC synthesized and proceeds through a stable peptidyl-succinimide intermediate nucleoside (5). While the peptide parts of different McCs differ greatly in their sequences and lengths (although the C-terminal asparagine is strictly conserved), their nucleotide parts so far are found in only two distinct forms, namely adenosine or cytosine monophosphates with or without additional decorations (4, 6) (Fig. 1B).

The E. coli McC (McC^Eco^) inhibits the growth of E. coli and closely related bacteria lacking the *mcc* operon by using a Trojan horse mechanism (7). The compound is actively imported into the target cell through the oligopeptide transporter YejABEF (8). In the cytoplasm, the 7-amino-acid-long peptide part is proteolytically processed by aminopeptidases PepA, PepB, and PepN with the release of the active payload—isoasparaginyl-AMP decorated with an aminopropyl group at the phosphate (9). This “processed McC^Eco^” is a structural mimic of aspartyl-adenosyl monophosphate, an intermediate of the tRNA^Asp^ charging reaction catalyzed by aspartyl-tRNA synthetase (AspRS). Binding of processed McC in the active center of AspRS results in protein biosynthesis inhibition (7), stringent response (10), and, eventually, cell death. All McC-like compounds studied to date have the Trojan horse mechanism of action and target AspRS (7, 11). The conservation of essential genes in *mcc* clusters and the fact that all MccA precursor peptides contain a terminal asparagine residue imply that both the mechanism and the intracellular target are conserved for all compounds of this class. Indeed, cytosine-containing McC-like compounds also target the AspRS (6, 11).

While mature McC-like compounds are exported from the producing cell by dedicated transporters, a certain amount is inevitably processed, releasing the toxic payload inside the producer (10). Several strategies to prevent self-intoxication of producers have been described. The Gcn5-related *N*-acetyltransferase MccE2 acetylates the primary amino group of processed McC^Eco^, thus preventing its binding to AspRS (12). l,d-Carboxypeptidase MccF hydrolyses the carboxamide bond between the peptide and nucleotide parts of intact McC^Eco^ or aminoacyl and the nucleotide parts of processed McC (13). Phosphoramidase MccH from Hyalangium minutum hydrolyzes the phosphoramide linkage of processed McC (14).

In this study, we report a new protein from Nocardia vaccinii *mcc*-like gene cluster that provides immunity to McC-like compounds. We name this protein MccG. MccG belongs to a superfamily of S51 peptidases. The type member of this family is peptidase E (PepE)—an aspartyl dipeptidase that hydrolyzes the peptide bond in dipeptides with an N-terminal l-aspartate (15). Here, we show that MccG from Nocardia vaccinii and its homologs hydrolyzes the carboxamide bond in processed forms of McC-like compounds with the formation of aspartate and a modified nucleotide phosphoramide. We further show that MccG homologs frequently encoded by standalone genes in various bacterial genomes can protect cells from McC-like compounds. Our findings reveal a new strategy of resistance to toxic aminoacyl-nucleotides and underscore the diversity of strategies that have been harnessed in the course of evolution to address the problem of self-intoxication of producers of McC-like RIPPs.

## RESULTS

**FIG 2:**
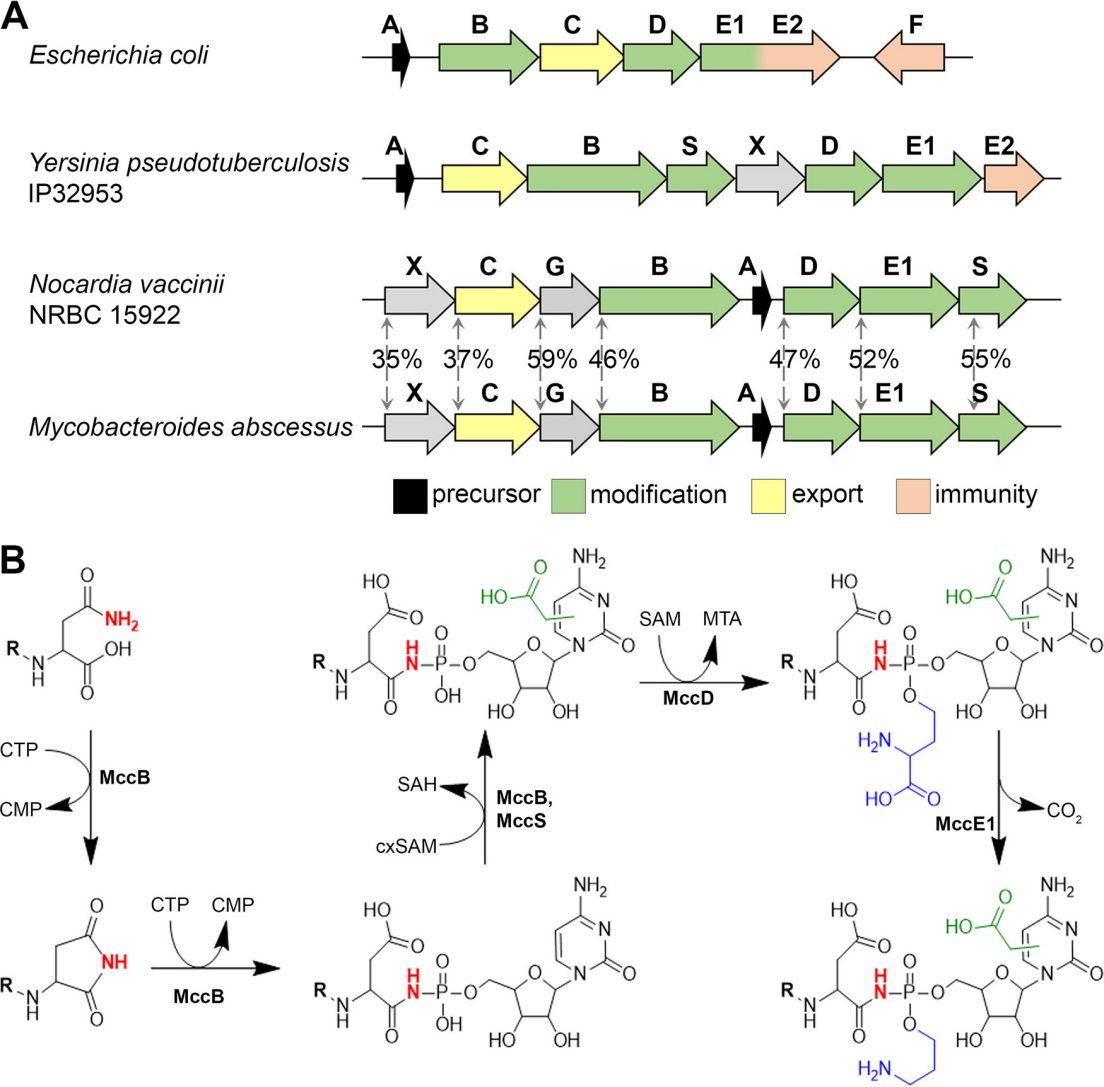
The *mcc*-like cluster from *N. vaccinii* NBRC 15922 (locus tags NV1_RS35535 to NV1_RS35560) and its products. (A) Organization of the *mcc*-like biosynthetic gene clusters (BGCs) from E. coli, Y. pseudotuberculosis IP 32953, *N. vaccinii* NBRC 15922, and M. abscessus subsp. *massiliense*. The general functions of the genes are indicated by colors and are discussed in the text. Arrows with the numbers between aligned BGCs indicate the degree of identity of amino acid sequences of *N. vaccinii* and M. abscessus *mcc* gene products. (B) Proposed mechanism of posttranslational modifications of MccA^Nva^. Carboxymethyl and the (carboxy)aminopropyl groups are shown in green and blue, respectively, and the nitrogen atom involved in the phosphoramide bond is shown in red.

During bioinformatical screening of sequenced bacterial genomes, we identified a group of *mcc-*like biosynthetic gene clusters (BGCs) in Nocardia vaccinii NBRC 15922 and various Mycobacteroides abscessus strains (2). Despite some differences in their architecture, these clusters closely resemble the previously characterized *mcc* operon from Yersinia pseudotuberculosis IP32953 (11) (Fig. 2A). We decided to focus our study on N. vaccinii *mcc*.

The *mcc* cluster from *N. vaccinii* contains a putative *mccA* gene, which codes for a 21-amino-acid-long precursor peptide, and *mccB^Nva^*, which codes for a protein similar to Yersinia pseudotuberculosis MccB (MccB^Yps^). Unlike the E. coli nucleotidyltransferase MccB, MccB^Yps^ and MccB^Nva^ are bifunctional proteins containing an N-terminal nucleotidyltransferase domain and a C-terminal carboxymethyl transferase domain. The product of *mccS^Nva^* is homologous to MccS^Yps^, a carboxy-*S*-adenosylmethionine (cxSAM) synthase (11). The products of *mccD^Nva^* and *mccE_1_^Nva^* are homologous to enzymes that jointly install the aminopropyl decoration at the phosphate group of peptidyl nucleotides in E. coli and Y. pseudotuberculosis (11, 16). A homolog of *mccX^Yps^*, which encodes a protein of unknown function, is also present in the *N. vaccinii mcc* operon. The *N. vaccinii mcc* cluster contains an additional gene, *mccG^Nva^*, located between the *mccC* and *mccB* genes. Such a gene is absent from the Y. pseudotuberculosis *mcc* operon (Fig. 2A) but is present in the identical location of the M. abscessus clusters.

By analogy with Y. pseudotuberculosis microcin C (McC^Yps^) one can assume that *N. vaccinii* McC (McC^Nva^) comprises the MccA peptide modified with carboxymethylated cytidylate and additionally decorated with an aminopropyl moiety (Fig. 2B). To test this hypothesis, we partially reconstructed the proposed pathway *in vitro* using the synthetic MccA^Nva^ precursor and the recombinant MccB^Nva^ (see Fig. S1 in the supplemental material). Matrix-assisted laser desorption ionization mass spectrometry (MALDI-MS) analysis of the reaction products allowed identification of MccA^Nva^-cytidylate and MccA^Nva^-carboxymethylated cytidylate, thus confirming that MccA^Nva^ is cytidylated and carboxymethylated by MccB^Nva^.

10.1128/mbio.00805-22.1 FIG S1 (A) Matrix-assisted laser desorption ionization (MALDI) mass spectrometry (MS) spectra of synthetic MccA^Nva^ precursor peptide (upper) and the products of its *in vitro* modification by recombinant MccB^Nva^ (lower). The *in vitro* coupled nucleotidylation-carboxymethylation reaction was performed in the presence of a chemically synthesized carboxy-*S*-adenosylmethionine (cxSAM) and an equimolar mix of four nucleoside triphosphates (NTPs) (11). [M+H]^+^ at *m/z* 2,305.5 corresponds to unmodified MccA^Nva^; mass ions at *m/z* 2,610.5 and at *m/z* 2,668.5 match MccA^Nva^-cytidylate and carboxymethylated MccA^Nva^-cytidylate, respectively. [M+Na]^+^ at *m/z* 2,632.5, marked with an asterisk, corresponds to a sodium adduct of MccA^Nva^-cytidylate. (B) MALDI-TOF tandem mass spectrometry (MS/MS) spectrum of the products of MccA^Nva^ *in vitro* modification by MccB^Nva^ ([M+H]^+^ ions at *m/z* 26,68.5). Mass shifts for 44 and 58 Da correspond to removal of carboxyl and carboxymethyl groups, respectively. Mass differences at 111 and 194 Da match the losses of cytosine nucleobase and monophosphorylated ribose, correspondingly. The [M+H]^+^ ion at *m/z* 2,287 matches the dehydrated MccA peptide (MKIVLKLKRIVRGAGPIIVSN). Download FIG S1, PDF file, 0.04 MB. Copyright © 2022 Yagmurov et al. 2022 Yagmurov et al. https://creativecommons.org/licenses/by/4.0/ This content is distributed under the terms of the Creative Commons Attribution 4.0 International license.

The Y. pseudotuberculosis *mcc* operon encodes MccE_2_^Yps^, a self-immunity enzyme that acetylates the amino group of processed McC, making it unable to inhibit AspRS. No such enzyme is encoded by the *N. vaccinii mcc* operon. In fact, no genes coding for any known immunity proteins are present in *N. vaccinii mcc*. We therefore hypothesized that the product of *mccG* gene, a gene that is absent in previously characterized *mcc* operons, can perform the immunity function in the *N. vaccinii mcc* BGC. MccG^Nva^ belongs to the MEROPS S51 family of peptidases (17), which includes PepE from Salmonella enterica (18) and CphB cyanophycinase from Synechocystis sp. PCC6803 (19), which hydrolyze Asp-Xaa dipeptides and multi-l-arginyl-poly-(l-aspartic acid) cyanophycin, respectively. We hypothesized that the product of the *mccG^Nva^* gene encodes a peptidase that cleaves peptidyl-nucleotides.

To test the proposed immunity function, we cloned *mccG^Nva^* into an arabinose-inducible pBAD vector and transformed the resulting plasmid, pBAD-*mccG^Nva^*, into McC-susceptible E. coli cells. Cells transformed with the pBAD-*mccF^Eco^* plasmid expressing the self-immunity l,d-carboxypeptidase MccF from E. coli *mcc* BGC were used as a positive control. Cells harboring an empty pBAD vector served as a negative control.

**FIG 3:**
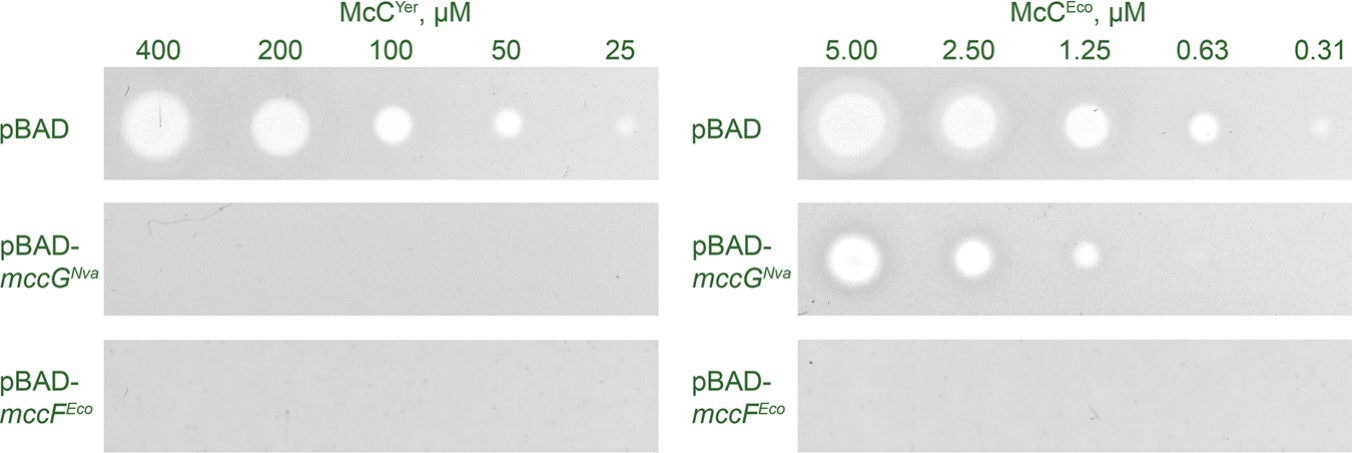
Overexpression of *mccG^Nva^* renders E. coli cells resistant to peptidyl-nucleotides. Drops of McC^Yps^ and McC^Eco^ solutions at the indicated concentrations were deposited on the surface of freshly seeded lawns of McC-susceptible E. coli cells transformed with the indicated plasmids. Photographs of plates after overnight growth at 30°C under conditions of induction of cloned plasmid-borne genes are shown.

To check if E. coli cells expressing *mccG^Nva^* acquired resistance to peptidyl-nucleotides, drops of solution of McC^Eco^ and McC^Yps^ were deposited on the surface of freshly seeded cell lawns, and the formation of growth inhibition zones was monitored after overnight incubation of plates at 30°C (see Materials and Methods). As can be seen from Fig. 3, both McC^Yps^ and McC^Eco^ inhibited the growth of E. coli cells harboring the empty vector. In contrast, expression of *mccF^Eco^* provided resistance to both McCs, as expected. Cells containing pBAD-*mccG^Nva^* were fully resistant to McC^Yps^ and partially resistant to McC^Eco^. We conclude that MccG^Nva^ is indeed a self-immunity enzyme capable of inactivating modified peptidyl-nucleotides, with a higher apparent preference toward peptidyl-cytidylates.

To identify the mechanism of immunity provided by MccG^Nva^, the recombinant MccG^Nva^ and MccF^Eco^ proteins were incubated with McC^Yps^ and McC^Eco^. The reaction products were separated using reversed-phase high-performance liquid chromatography (RP-HPLC), and the content of chromatographic peaks was analyzed with high-resolution electrospray ionization (ESI)-MS. No changes to the original compounds were observed when McC^Yps^ and McC^Eco^ were incubated with MccG^Nva^ (Fig. S2). In contrast, MccF^Eco^ efficiently cleaved off the peptide parts of both McC^Yps^ and McC^Eco^, as confirmed by accumulation of the [M+H]^+^ mass ions at *m/z* 438.14 and 404.14, respectively (Fig. S2). We therefore conclude that mature McC^Yps^ and McC^Eco^ are not substrates for MccG^Nva^.

10.1128/mbio.00805-22.2 FIG S2 Intact McC-like compounds are not substrates for MccG^Nva^. Reversed-phase high-performance liquid chromatography (RP-HPLC) elution profiles of McC^Yps^ ([M+2H]^2+^ at *m/z* 658.2061, [M+3H]^3+^ at *m/z* 439.1395), McC^Eco^ ([M+2H]^2+^ at *m/z* 589.2312, and [M + 3H]^3+^ at *m/z* 393.1566) alone or after incubation with MccG^Nva^ and MccF^Eco^. Download FIG S2, PDF file, 0.07 MB. Copyright © 2022 Yagmurov et al. 2022 Yagmurov et al. https://creativecommons.org/licenses/by/4.0/ This content is distributed under the terms of the Creative Commons Attribution 4.0 International license.

**FIG 4:**
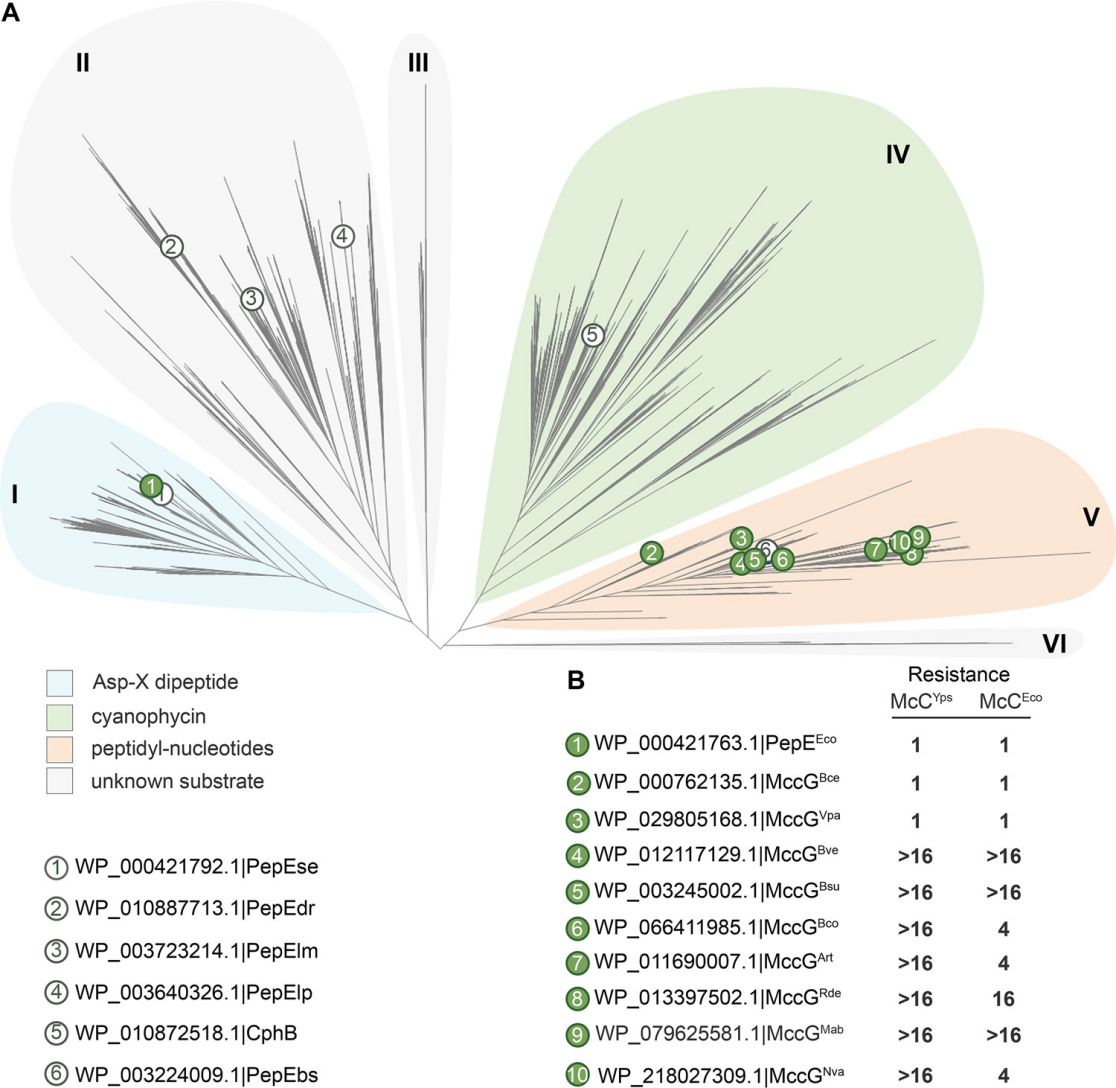
Phylogenetic analysis of MccG and its homologs from different bacteria. (A) Phylogenetic tree of aspartyl dipeptidases. Previously characterized representatives of S51 family peptidases (15, 19, 20) are numbered from 1 through 6 and highlighted with open circles. The *N. vaccinii* MccG (marked by a green circle and number 10) is located in a distinct clade, marked as V. Several MccG homologs from clade V are numbered from 1 through 9 and highlighted with green circles. (B) Resistance of McC-susceptible E. coli cells overproducing the indicated plasmid-borne MccG homologs to McC^Yps^ and McC^Eco^. Resistance was calculated as a ratio of MIC values for McC^Yps^ and McC^Eco^ obtained on lawns of E. coli cells transformed with plasmids expressing an MccG homolog to the MIC value obtained on lawns of cells transformed with an empty pBAD vector.

To infer the mechanism of protective activity of MccG^Nva^, a phylogenetic tree of S51 family peptidases was constructed using 3,097 protein sequences found in 13,116 completely sequenced archaeal and bacterial genomes. The resulting tree contains six major clades (Fig. 4). The previously studied PepE aspartyl dipeptidase from Salmonella enterica (18) and cyanophycinase form *Synechocystis* sp. PCC6803 (19) belong to clades I and IV, respectively (Fig. 4A). Several proteins that do not possess peptidase activity and have no established physiological function belong to clade II (Fig. 4A) (20). MccG^Nva^, together with PepE^Bs^ from Bacillus subtilis (20), belongs to clade V. PepE^Bs^ was previously shown to hydrolyze the model substrate of aspartyl dipeptidases, l-aspartic acid α-(*p*-nitroanilide) (Asp-*p*NA), *in vitro* (20); however, its natural substrates are unknown.

To check if the ability to confer resistance to McC-like compounds is unique to MccG^Nva^, we tested MccG^Mab^ from the M. abscessus *mcc* BGC and several MccG^Nva^ homologs from clade V encoded by standalone genes. The latter included MccG-like proteins from Arthrobacter sp. FB24, Bacillus cohnii DSM6307, Bacillus cereus ATCC 4342, Bacillus subtilis 168, Bacillus vallismortis DSM11031, Bacillus velezensis FZB42, Rothia dentocariosa ATCC 17931, and Vibrio parahaemolyticus ATCC 4342 (Fig. 4A). The genes encoding MccG^Nva^ homologs were cloned into the pBAD vector, and the resulting plasmids were transformed in McC-susceptible E. coli; cells expressing *mccG* homologs were tested for susceptibility to McC^Eco^ and McC^Yps^. Cells overexpressing cloned E. coli *pepE* were also tested.

As can be seen from Fig. 4B and Fig. S3, overexpression of E. coli dipeptidase PepE did not protect cells from McC action. To confirm that recombinant PepE is an active aspartyl dipeptidase, we synthesized its substrate, Asp-*p*NA (20). Incubation of Asp-*p*NA with recombinant PepE led to hydrolysis of the compound at the carboxamide bond and formation of chromogenic *p*-nitroaniline (Fig. S4). Thus, recombinant PepE^Eco^ is active but does not confer resistance to McC-like antibiotics.

10.1128/mbio.00805-22.3 FIG S3 Susceptibility to McC^Yps^ and McC^Eco^ of Escherichia coli harboring plasmids with the indicated *mccG^Nva^* homologs. Download FIG S3, PDF file, 0.2 MB. Copyright © 2022 Yagmurov et al. 2022 Yagmurov et al. https://creativecommons.org/licenses/by/4.0/ This content is distributed under the terms of the Creative Commons Attribution 4.0 International license.

10.1128/mbio.00805-22.4 FIG S4 Recombinant PepE^Eco^ hydrolyzes synthetic Asp-*p*NA substrate. Absorbance spectra of Asp-pNA (left) and Asp-pNA incubated with PepE^Eco^ (right). Download FIG S4, PDF file, 0.08 MB. Copyright © 2022 Yagmurov et al. 2022 Yagmurov et al. https://creativecommons.org/licenses/by/4.0/ This content is distributed under the terms of the Creative Commons Attribution 4.0 International license.

As expected, MccG^Mab^, which is encoded in an *mcc* gene cluster, conferred immunity to both McC^Yps^ and McC^Eco^. Interestingly, five out of seven tested MccG^Nva^ homologs that are encoded by standalone genes also provided resistance to McC^Yps^ and McC^Eco^. Expression of M. abscessus MccG^Mab^, B. subtilis 168 MccG^Bsu^, and B. velezensis MccG^Bve^ provided higher levels of resistance to McC^Eco^ than did MccG^Nva^. It remains to be determined whether this result is due to broader specificity of these enzymes or is a trivial effect of higher levels of production in a heterologous system. Two proteins belonging to the basal branches of clade V, MccG^Bce^ and MccG^Vpa^, did not confer immunity to McCs, suggesting that these enzymes have different substrate specificities than those of the rest of MccG enzymes tested.

**FIG 5:**
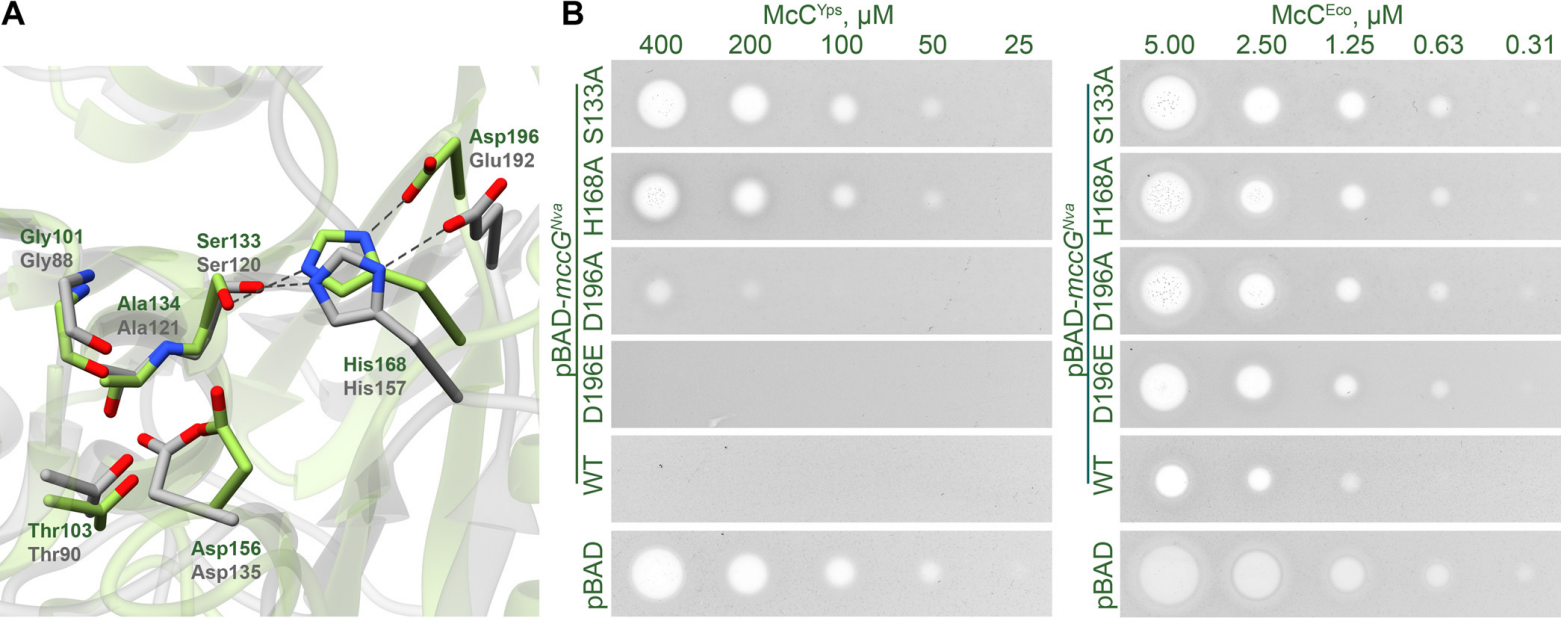
Mutational analysis of MccG^Nva^. (A) Superposition of the catalytic centers of PepE aspartyl dipeptidase (PDB accession number 6A4R; shown in gray) and the MccG^Nva^ structural model (shown in green). The catalytic residues are shown as sticks. (B) Resistance to McC^Yps^ and McC^Eco^ of E. coli cells overproducing the indicated MccG^Nva^ mutants.

To show that peptidase activity is required for the McC-protecting function of MccG^Nva^, we attempted to predict the amino acid residues important for catalysis. However, low sequence similarity between the S. enterica PepE, *Synechocystis* sp. CphB, and MccG^Nva^ precluded unambiguous identification of the catalytic triad (Fig. S5). We therefore performed structure modeling of MccG^Nva^ using AlfaFold v. 2.1.0 (21). The monomeric structure was modeled with high confidence (pLDDT > 90) for most of the protein sequence, except for 20 amino acids at the C terminus. As expected, the MccG^Nva^ showed the highest structural similarity to cyanophycinase from *Synechocystis* sp. PCC6803 (19) (PDB accession number 3EN0; root mean square deviation [RMSD] = 2.6) and PepE from S. enterica (22) (PDB accession number 6A4R; RMSD = 2.6). The superposition of the of S. enterica PepE and MccG^Nva^ monomers revealed an identical architecture of the catalytic center in two enzymes (Fig. 5A). The catalytic triad of PepE is composed of Ser120, His157, and Glu192 residues, which form a network of hydrogen bonds that position the nucleophilic Ser to interact with the substrate. However, while the catalytic Ser133 and His168 residues are conserved, the third amino acid in the MccG^Nva^ triad is an aspartate (Asp196). This residue is conserved in clade V enzymes.

10.1128/mbio.00805-22.5 FIG S5 A multiple sequence alignment of cyanophycinase from *Synechocystis* sp. (GenBank accession number WP_010872518.1), the PepE peptidase from Salmonella enterica (accession number WP_000421792.1), and MccG^Nva^ from Nocardia vaccinii NBRC 15922 (accession number WP_218027309.1) is presented. The alignment was built using MUSCLE with default parameters. Experimentally confirmed catalytic residues of Cph and PepE are highlighted in red. Based on the sequence alignment, only two of these residues, a serine and a histidine, are conserved in all three proteins. Download FIG S5, PDF file, 0.04 MB. Copyright © 2022 Yagmurov et al. 2022 Yagmurov et al. https://creativecommons.org/licenses/by/4.0/ This content is distributed under the terms of the Creative Commons Attribution 4.0 International license.

We used site-specific mutagenesis to substitute each of the conserved amino acid of MccG^Nva^ catalytic triad (Ser133, His168, and Asp196) for alanine. We have also reconstructed the “canonical PepE catalytic triad” in MccG^Nva^, by constructing a D196E single-substitution mutant with an expectation that this may affect its ability to cleave processed McCs, which PepE lacks. E. coli cells expressing the plasmid-borne mutant *mccG^Nva^* were tested for susceptibility to McC^Eco^ and McC^Yps^. Expectedly, Ser133Ala and His168Ala substitutions abolished MccG-mediated protection from McC^Yps^. Expression of MccG^Nva^ with the D196A substitution led to substantially decreased resistance to peptidyl-nucleotide antibiotics, while the D196E mutant was resistant to Mcc^Yps^ (Fig. 5B). Compared to wild-type MccG^Nva^, the ability of D196E mutant to protect cells from McC^Eco^ was somewhat compromised, as judged by the size of growth inhibition zones. We conclude that the catalytic activity of MccG^Nva^ is required for protecting cells against inhibitory action of peptidyl-nucleotide antibiotics; however, the presence of an aspartate instead of glutamate found in PepE is not the sole determinant of MccG ability to detoxify processed McC.

**FIG 6:**
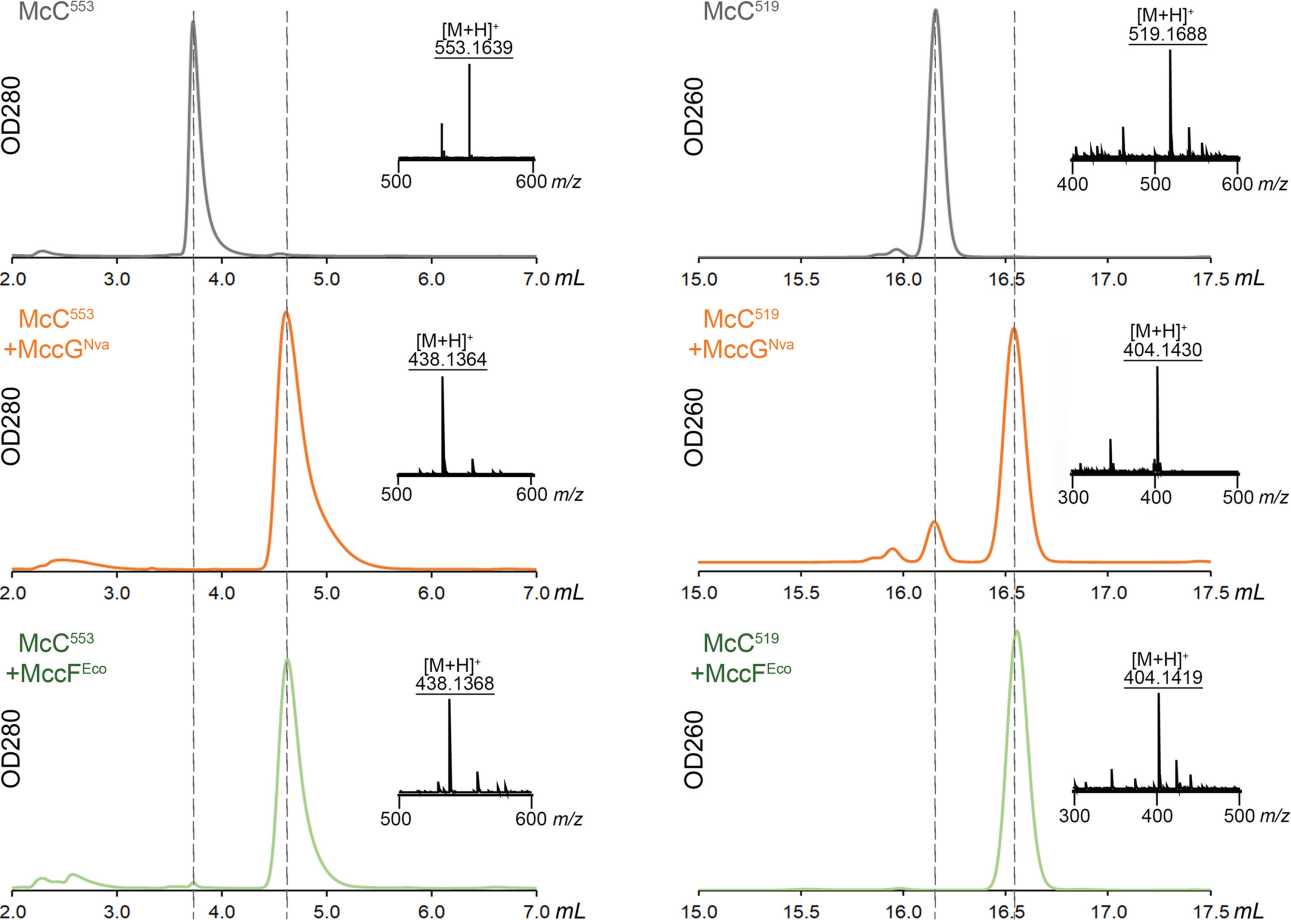
MccG^Nva^ hydrolyzes the carboxamide bond in isoasparginyl-carboxymethylcytidylate McC^553^ and isoasparaginyl-adenylate McC^519^. Reversed-phase high-performance liquid chromatography (RP-HPLC) elution profiles of the reaction products upon incubation of McC^553^ and McC^519^ without additional enzymes or with MccG^Nva^ or MccF^Eco^. Electrospray ionization mass spectrometry (ESI-MS) analyses of corresponding chromatographic peaks are superimposed with HPLC elution profiles. [M+H]^+^ at *m/z* 553.1632 corresponds to McC^553^; [M+H]^+^ at *m/z* 519.1688 corresponds to McC^519^; [M+H]^+^ at *m/z* 438.1384 corresponds to aminopropylated carboxymethylcytidine phosphoramide; and [M+H]^+^ at *m/z* 404.1449 corresponds to aminopropylated adenosine phosphoramide.

We next hypothesized that MccG^Nva^ is a peptidase capable of hydrolyzing the peptide bond solely in processed McCs, small molecules with an aspartate residue coupled to a nucleotide. We prepared aminopropylated forms of carboxymethylated aspartamide-cytidylate (McC^553^) and aspartamide-adenylate (McC^519^) by *in vitro* proteolysis of mature McC^Yps^ and McC^Eco^, respectively, and incubated them with recombinant MccG^Nva^ or MccF^Eco^. The reaction products were separated by RP-HPLC and subjected to ESI-MS analysis. Both MccF^Eco^ and MccG^Nva^ fully converted McC^553^ into a new compound with distinct chromatographic mobility. The ESI-MS analysis of the observed peak revealed a [M+H]^+^ ion at *m/z* 438.1364, matching aminopropylated carboxymethylcytidine phosphoramide (calculated monoisotopic mass of the ion is 438.1384 Da) (Fig. 6). The tandem mass spectrometry (MS/MS) fragmentation spectra of the compound verified the assignment (Fig. S6).

10.1128/mbio.00805-22.6 FIG S6 Electrospray ionization (ESI) tandem mass spectrometry (MS/MS) fragmentation spectra of the products of MccG^Nva^-mediated hydrolysis of McC^519^ (upper) and McC^553^ (lower). Parent ions on mass spectra are labeled in red. Download FIG S6, PDF file, 0.04 MB. Copyright © 2022 Yagmurov et al. 2022 Yagmurov et al. https://creativecommons.org/licenses/by/4.0/ This content is distributed under the terms of the Creative Commons Attribution 4.0 International license.

After incubation of McC^519^ with MccG^Nva^ the chromatographic peak corresponding to the initial compound ([M+H]^+^ ion at *m/z* 519.1685) was observed alongside with a ([M+H]^+^ ion at *m/z* 404.1419). The conversion was complete in reaction mixtures containing MccF^Eco^. The [M+H]^+^ ion at *m/z* 404.1419 matches aminopropylated adenosine phosphoramide. ESI MS/MS spectrum of the compound confirmed the assignment (Fig. S6). Thus, MccG^Nva^ hydrolyzes carboxamide bond in processed McC-like with a clear preference toward aminoacyl cytidylates.

Given the data presented above, we conclude that MccG^Nva^ hydrolyzes the carboxamide bond in processed McC-like compounds through a mechanism that is similar to that used by serine proteases to hydrolyze peptide bonds (Fig. S7).

10.1128/mbio.00805-22.7 FIG S7 Proposed reaction mechanism of MccG. At the first step, Ser133, deprotonated with His168, attacks the carbonyl group of processed McC aspartate, forming the first tetrahedral complex. The His168 complex with proton is stabilized via a hydrogen bond with the negatively charged Asp196. The His168 hydrogen is then taken by the nitrogen of the phosphamide bond of the substrate, releasing the nucleotide part. Next, the remaining covalently bound aspartate is attacked by a water molecule, which, similarly to Ser133 at the first step, is deprotonated by His168. The newly formed tetrahedral complex decomposes with the release of free aspartate, and the catalytic triad is returned to its initial state. Download FIG S7, PDF file, 0.09 MB. Copyright © 2022 Yagmurov et al. 2022 Yagmurov et al. https://creativecommons.org/licenses/by/4.0/ This content is distributed under the terms of the Creative Commons Attribution 4.0 International license.

## DISCUSSION

The mechanism of McC-like compounds action necessitates the need for immunity strategies against the compound, whether endogenously produced, or imported from the outside. While minimal *mcc* operons containing just three genes coding for the peptide precursor—the nucleotidyl transferase that produced the active compound, and the export pump—are known, many *mcc*-like gene clusters encode enzymes that provide self-immunity to the producing cell (2, 3). Using *in vitro* reconstitution, we show here that the product of Nocardia vaccinii *mcc* is a peptidyl-cytidylate similar to McC^Yps^ (see Fig. S1 in the supplemental material) (11). In Y. pseudotuberculosis *mcc*, self-immunity is achieved through the function of MccE2, which covalently modifies processed McC rendering it inactive. In this paper, we describe a novel self-immunity enzyme MccG from the *N. vaccinii mcc* operon. We show that MccG is an unusual peptidase that hydrolyzes the carboxamide bond between the aminoacyl and nucleotide moieties of processed McC (Fig. 3). MccG homologs encoded by standalone genes can also protect cells from McC.

It is not clear at this point which of the two mechanisms of self-immunity is ancestral. While *mccE* genes are found in different locations in *mcc* operons from different sources, the *mccG* genes known so far are found in the *N. vaccinii* and M. abscessus *mcc* BGCs and their location is distinct from known locations of *mccE* genes. Be that as it may, comparisons of different BGCs suggest that self-immunity functions can be lost from *mcc* operons and analogous functions can then be regained by recruiting standalone genes from the core genome of the bacterial cell. While this has not been explicitly checked, it is possible that minimal three-gene *mcc* operons reside in bacteria which provide the self-immunity functions by standalone MccE_2_-, MccG-, MccH-, and MccF-like enzymes that mop up toxic processing products of *mcc* operons accumulating inside the producers, whether from intracellularly produced compounds or from those imported from the outside. Recruitment of any one of these genes or their combinations provides a clear advantage for mobile genetic elements that often carry *mcc* operons and allow them to efficiently colonize diverse hosts without decreasing their fitness.

Unlike MccF, a previously described McC self-immunity peptidase (12), MccG^Nva^ can only detoxify processed forms of McC-like compounds (Fig. 4 and Fig. S2). While MccF cleaves both modified peptidyl-adenylates and cytidylates efficiently, MccG from *N. vaccinii* has a clear preference for peptidyl-cytidylates (Fig. 4). Yet, M. abscessus MccG^Mab^, B. subtilis 168 MccG^Bsu^, and *B. velezensis* MccG^Bsu^ provided higher levels of resistance than MccG^Nva^ to McC^Eco^. It is thus possible that some MccG homologs are as versatile as MccF and can protect cells from both cytosine and adenosine containing McC-like compounds.

## MATERIALS AND METHODS

### DNA manipulation, molecular cloning, and protein purification.

All cloning steps were conducted in E. coli DH5α (F^−^ φ80*lacZ*ΔM15 Δ(*lacZYA-argF*) *U169 recA1 endA1 hsdR17*(r_K_^−^, m_K_^+^) *phoA supE44* λ^−^ *thi-1 gyrA96 relA1*). Protein purification was performed in E. coli BL21(DE3) (F^−^ *ompT hsdSB* [r_B_^−^, m_B_^−^] *gal dcm* [DE3]). Phusion high-fidelity DNA polymerase (Thermo Scientific, USA) was used for DNA amplification. DNA primer synthesis and DNA sequencing were performed by Evrogen (Russia). For the list of primers used in the study, refer to Table S1 in the supplemental material. Unless indicated otherwise, the genomic DNA was used as a template for PCR amplification.

10.1128/mbio.00805-22.8 TABLE S1 Primers used in the study. Download Table S1, DOCX file, 0.02 MB. Copyright © 2022 Yagmurov et al. 2022 Yagmurov et al. https://creativecommons.org/licenses/by/4.0/ This content is distributed under the terms of the Creative Commons Attribution 4.0 International license.

For production of recombinant MccB^Nva^ enzyme, PCR-amplified *mccB^Nva^* gene was digested with BamHI and SacI restriction endonucleases and introduced into pRSFDuet-1 vector digested with the same endonucleases. The resultant pRSF_6×His_*mccB^Nva^* contained a sequence encoding MccB^Nva^ enzyme fused with an N-terminal hexahistidine affinity tag.

For *in vivo* spot toxicity assay, pBAD_SalRBS (14) vector was digested by SalI and HindIII and combined with the PCR-amplified fragments of the corresponding genes digested with same restriction endonucleases. Genes encoding MccG homologs from Rothia dentocariosa ATCC 17931, Mycobacteroides abscessus subsp. *massiliense* strain 616, and Vibrio parahaemolyticus ATCC 17802 were purchased from IDT (USA) as synthetic DNA fragments. To create mutants of MccG^Nva^, site-directed mutagenesis was employed using overlap extension PCR (23).

For recombinant protein purification of MccF^Eco^, PepE^Eco^, MccG^Nva^ its homologs and mutants, the PCR-amplified genes encoding corresponding enzymes and lacking a stop codon were digested with NdeI and XhoI and introduced into a linearized (with the same restriction endonucleases) pET22 vector (Novagen-Millipore, USA) with an engineered sequence encoding a C-terminal hexahistidine affinity tag.

For protein production, the E. coli BL21(DE3) strain transformed with an appropriate plasmid was grown in 250 mL of TB medium supplemented with kanamycin at 37°C, and moderate constant shaking until an optical density at 600 (OD_600_) of ∼0.6. Upon reaching an OD_600_ of ∼0.6, the bacterial culture was induced with 0.5 mM isopropyl-β-d-thiogalactopyranoside (IPTG) and grown at 18°C for an additional 16 h. The cells were harvested by centrifugation, resuspended in buffer A (20 mM Tris-HCl ([pH 8.0], 150 mM NaCl, 2 mM imidazole, and 5% glycerol), supplemented with 0.5 mM phenylmethylsulfonyl fluoride (PMSF) and disrupted by sonication. The resultant lysate was cleared by centrifugation at 30,000 × *g* for 20 min and applied to Talon CellThru Co^2+^ chelating resin (TaKaRa-Clontech). The protein-bound resin was washed with 3 column volumes (CV) of buffer A and 5 CV of buffer B (20 mM Tris-HCl [pH 8.0], 50 mM NaCl, 20 mM imidazole). The protein was eluted with 2 CV of buffer C (20 mM Tris-HCl [pH 8.0], 50 mM NaCl, 300 mM imidazole, and 10% glycerol). The fractions containing the eluted protein were analyzed by SDS-PAGE and bands corresponding to purified proteins were subjected to tryptic digestion with subsequent matrix-assisted laser desorption ionization–time of flight (MALDI-TOF) mass spectrometry identification of the protein. Following elution, the protein was dialyzed in buffer D (20 mM Tris-HCl [pH 8.0], 50 mM NaCl, and 10% glycerol), and stored at −80°C until further use.

### *In vitro* nucleotidylation and carboxymethylation reactions.

To recreate the McC^Nva^ biosynthesis *in vitro*, the nucleotidyl transfer and carboxymethylation reactions were conducted using the synthetic MccA^Nva^ peptide (MKIVLKLKRIVRGAGPIIVSN), chemically synthesized cxSAM (6, 24) and a recombinant MccB^Nva^ protein. The reaction mixture containing 100 μM chemically synthesized MccA^Nva^ peptide, 2 mM equimolar mix of nucleotide triphosphates, 2 mM cxSAM, and 5 μM recombinant MccB^Nva^ in a reaction buffer (50 mM Tris-HC [pH 8.0] 150 mM NaCl, 5 mM MgCl_2_, and 5 mM dithiothreitol [DTT]) was incubated for 16 h at 28°C, then stopped by addition of trifluoroacetic acid (TFA) to a concentration of 0.1% (vol/vol). The products of the reaction were analyzed by MALDI-TOF mass spectrometry for the presence of peptidyl-carboxymethylcytidine.

### *In vivo* McC toxicity assay.

E. coli strain B was transformed with the pBAD_SalRBS vector containing *mccF^Eco^*, *pepE^Eco^*, or *mccG^Nva^* or its homologs. Transformants were selected on LB plates containing 100 μg/mL ampicillin. A single colony was inoculated into 2× yeast extract-tryptone (YT) medium supplemented with 100 μg/mL ampicillin and 10 mM arabinose and grown at 37°C for 16 h. The overnight culture was diluted 1,000-fold in M9 agar medium, supplemented with 1% glycerol, 0.1% yeast extract, 10 mM arabinose, and 100 μg/mL ampicillin. Drops (3 μL) of McC^Eco^ and McC^Yps^ of various concentrations were placed on the surface of the agar plate and allowed to dry. Plates were incubated for 16 h at 30°C to form a lawn.

### Preparation of McC^553^ and McC^519^.

Purification of McC^Eco^ and McC^Yps^ was performed as described previously (11). HPLC-purified McC^Yps^ and McC^Eco^ were dissolved in 3 mL of the reaction buffer [50 mM HEPES (pH 7.4) 0.75 mM MnCl_2_, and 5 mM Tris(2-carboxyethyl)phosphine (TCEP)] to a concentration of 100 μM. The solution was then combined with recombinant PepA, PepB, and PepN from E. coli to a concentration of 10 μM each, and the mixture was incubated at 32°C for 16 h. Upon incubation, the reaction was quenched by the addition of an equal volume of 100% acetonitrile. The pH of the solution was adjusted to 2.0 by addition of TFA, and the solution was incubated for 30 min on ice to allow the proteins to precipitate. After incubation, the protein precipitate was removed by centrifugation at 16,000 × *g* for 15 min (4°C), and the supernatant was collected and lyophilized. The protein pellet was then extracted 2 times with a 50% acetonitrile solution in deionized water and the extracts were also lyophilized.

The supernatant and extract fractions were dissolved in 0.1% TFA solution in deionized water and applied to a Prep 5 C_18_ column (10 mm by 250 mm, particle size = 5 μm; Agilent Technologies). The processed McC^Yps^ and McC^Eco^ were first purified in a 0.1% TFA-acetonitrile system in a linear 1 to 13% gradient of acetonitrile, followed by a second round of purification in ammonium acetate buffer (pH 4.3) in a linear 1 to 13% gradient of acetonitrile on a Triart C_18_ column (150 mm by 3 mm, particle size = 3 μm; YMC).

### Chemical synthesis of Asp-*p*NA.

Asp-*p*NA was synthesized from protected fluorenylmethoxycarbonyl protecting group (Fmoc)-Asp(Ot-Bu)-OH (Sigma) by coupling with 4-niroaniline (*p*NA) (Sigma) as described by Nedev et al. (25) with minor modifications. Briefly, Fmoc-Asp(Ot-Bu)-OH was activated with isobutyl chloroformate in tetrahydrofuran (THF), which facilitated a subsequent reaction with weakly nucleophilic 4-nitroaniline. Fmoc deprotection was performed with Tris(2-aminoethyl)amine (TAEA) as described by Peterson et al. (26). The side-chain carboxyl group was deprotected from t-Bu in TFA-CH_2_Cl_2_ (19:1) for 30 min. Solvent was removed by rotary evaporation. Solid product was washed with ethyl either, lyophilized, resuspended in 0.1% TFA in Milli-Q water (MQ) and applied to a Agilent Prep preparative LC column (10 mm by 250 mm, particle size = 5 μm; Agilent Technologies). The purification of Asp-*p*NA was carried out in a linear 5 to 25% gradient of acetonitrile; fractions absorbing at 313 nm were analyzed for the presence of Asp-*p*NA by ESI mass spectrometry. The fractions containing a validated Asp-*p*NA were subjected to additional chromatographic purification on the same columns in a linear 15 to 20% gradient of acetonitrile in 30 mM ammonium acetate buffer (pH 4.3).

### *In vitro* hydrolysis assays.

To test the hydrolytic activities of MccF^Eco^, PepE^Eco^, and MccG^Nva^ and its homologs and mutants, 1 μM of each respective recombinant enzyme was combined with 100 μM substrate (either McC^Yps^, McC^Eco^ or their processed forms, McC^553^ and McC^519^, respectively) in reaction buffer (50 mM HEPES [pH 7.4], 2 mM MgCl_2_, 100 mM NaCl, and 2 mM DTT). The mixture was incubated for 30 min at 28°C, and then the reaction was quenched by addition of TFA to a concentration of 0.1% (vol/vol). The reaction products were analyzed by RP-HPLC, followed by ESI-MS analysis.

To test the peptidase E activity of enzymes, 200 μM Asp-*p*NA was combined with 2 μM recombinant enzyme in the reaction buffer (50 mM HEPES [pH 7.4], 2 mM MgCl_2_, 100 mM NaCl, and 2 mM DTT). The hydrolysis was carried out for 30 min at 28°C and then stopped by addition of TFA. The hydrolysis of Asp-*p*NA was monitored by the increase in optical density at OD_410_ corresponding to the accumulation of released *p*NA, using the NanoDrop 2000c spectrophotometer (Thermo Scientific, USA).

### RP-HPLC analysis of the products of *in vitro* reactions.

The analysis of enzymatic reactions was performed on an Infinity II 1260 liquid chromatography system (Agilent). Separation of the enzymatic reaction products occurred on a Triart C_18_ column (YMC) in 30 mM ammonium acetate buffer system (pH 4.3) in a linear gradient of acetonitrile from 0 to 10% for 25 min. Processing of chromatograms was performed in OpenLab CDS ChemStation (Agilent). The elution profiles were exported in comma-separated value (.csv) format for visualization.

### Mass spectrometry analysis.

**(i) High-resolution electrospray ionization mass spectrometry analysis.** Q-TOF Maxis Impact II (Bruker Daltonics) mass spectrometer with electrospray ionization was used for sample analysis. Lyophilized HPLC fractions from biochemical reactions were dissolved in 0.1% formic acid in deionized water and introduced directly to the instrument using the syringe pump. The spectra were recorded in a positive ion mode in a range from 100 *m/z* to 750 *m/z*. The temperature of ion source was 200°C, pressure of carrier gas 2.5 bar, the gas flow 5 mL/min, the voltage at the capillary 4 kV. The fragmentation spectra were recorded in AutoMS mode.

### (ii) Matrix-assisted laser desorption–time of flight mass spectrometry.

Sample aliquots were combined with the matrix mix (Sigma-Aldrich) on a steel target. The mass spectra were recorded on an UltrafleXtreme MALDI-tandem time of flight (TOF/TOF) mass spectrometer (Bruker Daltonics) equipped with a neodymium laser. The molecular MH^+^ ions were measured in reflector mode; the accuracy of the measured results was within 0.1 Da.

### Phylogenetic analysis of S51 family peptidases.

Sequence alignments of S51 family peptidases from the NCBI Conserved Domains Database (CDD) (CDD identifiers cd03129, cd03145, and cd03146) were used as queries in PSI-BLAST (27) searches against a database containing 21.4 million protein sequences encoded in 13,116 completely sequenced archaeal and bacterial genomes, available as of March 2019 (obtained from the NCBI FTP site, https://ftp.ncbi.nlm.nih.gov/genomes/ASSEMBLY_REPORTS/). Sequences matching these profiles were clustered using MMSEQS2 (28) with a similarity threshold of 0.5; sequences within each cluster were aligned using MUSCLE (29). Alignments were compared to each other using HHSEARCH (30) and iteratively merged using HHALIGN, guided by an unweighted pair group method with arithmetic mean (UPGMA) tree constructed from the matrix of HHSEARCH scores. The approximate maximum-likelihood tree was reconstructed using FastTree (31) with the WAG evolutionary model and gamma-distributed site rates.

## References

[1] Montalbán-López M, Scott TA, Ramesh S, Rahman IR, van Heel AJ, Viel JH, Bandarian V, Dittmann E, Genilloud O, Goto Y, Grande Burgos MJ, Hill C, Kim S, Koehnke J, Latham JA, Link AJ, Martínez B, Nair SK, Nicolet Y, Rebuffat S, Sahl H-G, Sareen D, Schmidt EW, Schmitt L, Severinov K, Süssmuth RD, Truman AW, Wang H, Weng J-K, van Wezel GP, Zhang Q, Zhong J, Piel J, Mitchell DA, Kuipers OP, van der Donk WA. 2021. New developments in RiPP discovery, enzymology and engineering. Nat Prod Rep 38:130–239. doi:10.1039/d0np00027b. 32935693 PMC7864896

[2] Travin DY, Severinov K, Dubiley S. 2021. Natural Trojan horse inhibitors of aminoacyl-tRNA synthetases. RSC Chem Biol 2:468–485. doi:10.1039/d0cb00208a. 34382000 PMC8323819

[3] Bantysh O, Serebryakova M, Makarova KS, Dubiley S, Datsenko KA, Severinov K. 2014. Enzymatic synthesis of bioinformatically predicted microcin C-like compounds encoded by diverse bacteria. mBio 5:e01059-14. doi:10.1128/mBio.01059-14. 24803518 PMC4010828

[4] Guijarro JI, González-Pastor JE, Baleux F, Millán JLS, Castilla MA, Rico M, Moreno F, Delepierre M. 1995. Chemical structure and translation inhibition studies of the antibiotic microcin C7. J Biol Chem 270:23520–23532. doi:10.1074/jbc.270.40.23520. 7559516

[5] Roush RF, Nolan EM, Löhr F, Walsh CT. 2008. Maturation of an *Escherichia coli* ribosomal peptide antibiotic by ATP-consuming N-P bond formation in microcin C7. J Am Chem Soc 130:3603–3609. doi:10.1021/ja7101949. 18290647

[6] Serebryakova M, Tsibulskaya D, Mokina O, Kulikovsky A, Nautiyal M, Van Aerschot A, Severinov K, Dubiley S. 2016. A Trojan-horse peptide-carboxymethyl-cytidine antibiotic from *Bacillus amyloliquefaciens*. J Am Chem Soc 138:15690–15698. doi:10.1021/jacs.6b09853. 27934031 PMC5152938

[7] Metlitskaya A, Kazakov T, Kommer A, Pavlova O, Ibba M, Kolb V, Severinov K, Metlitskaya A, Kazakov T, Kommer A, Pavlova O, Praetorius-Ibba M, Ibba M, Krasheninnikov I, Kolb V, Khmel I, Severinov K. 2006. Aspartyl-tRNA synthetase is the target of peptide nucleotide antibiotic microcin C. J Biol Chem 281:18033–18042. doi:10.1074/jbc.M513174200. 16574659

[8] Novikova M, Metlitskaya A, Datsenko K, Kazakov T, Kazakov A, Wanner B, Severinov K. 2007. The *Escherichia coli* Yej transporter is required for the uptake of translation inhibitor microcin C. J Bacteriol 189:8361–8365. doi:10.1128/JB.01028-07. 17873039 PMC2168686

[9] Kazakov T, Vondenhoff GH, Datsenko KA, Novikova M, Metlitskaya A, Wanner BL, Severinov K. 2008. *Escherichia coli* peptidase A, B, or N can process translation inhibitor microcin C. J Bacteriol 190:2607–2610. doi:10.1128/JB.01956-07. 18223070 PMC2293190

[10] Piskunova J, Maisonneuve E, Germain E, Gerdes K, Severinov K. 2017. Peptide-nucleotide antibiotic microcin C is a potent inducer of stringent response and persistence in both sensitive and producing cells. Mol Microbiol 104:463–471. doi:10.1111/mmi.13640. 28164379 PMC6876116

[11] Tsibulskaya D, Mokina O, Kulikovsky A, Piskunova J, Severinov K, Serebryakova M, Dubiley S. 2017. The product of *Yersinia pseudotuberculosis mcc* operon is a peptide-cytidine antibiotic activated inside producing cells by the TldD/E protease. J Am Chem Soc 139:16178–16187. doi:10.1021/jacs.7b07118. 29045133

[12] Agarwal V, Metlitskaya A, Severinov K, Nair SK. 2011. Structural basis for microcin C7 inactivation by the MccE acetyltransferase. J Biol Chem 286:21295–21303. doi:10.1074/jbc.M111.226282. 21507941 PMC3122189

[13] Tikhonov A, Kazakov T, Semenova E, Serebryakova M, Vondenhoff G, Van Aerschot A, Reader JS, Govorun VM, Severinov K. 2010. The mechanism of microcin C resistance provided by the MccF peptidase. J Biol Chem 285:37944–37952. doi:10.1074/jbc.M110.179135. 20876530 PMC2992228

[14] Yagmurov E, Tsibulskaya D, Livenskyi A, Serebryakova M, Wolf YI, Borukhov S, Severinov K, Dubiley S. 2020. Histidine-triad hydrolases provide resistance to peptide-nucleotide antibiotics. mBio 11:e00497-20. doi:10.1128/mBio.00497-20. 32265328 PMC7157772

[15] Conlin CA, Hakensson K, Liljas A, Miller CG. 1994. Cloning and nucleotide sequence of the cyclic AMP receptor protein- regulated *Salmonella typhimurium pepE* gene and crystallization of its product, an α-aspartyl dipeptidase. J Bacteriol 176:166–172. doi:10.1128/jb.176.1.166-172.1994. 8282693 PMC205028

[16] Kulikovsky A, Serebryakova M, Bantysh O, Metlitskaya A, Borukhov S, Severinov K, Dubiley S. 2014. The molecular mechanism of aminopropylation of peptide-nucleotide antibiotic microcin C. J Am Chem Soc 136:11168–11175. doi:10.1021/ja505982c. 25026542

[17] Rawlings ND, Barrett AJ, Thomas PD, Huang X, Bateman A, Finn RD. 2018. The MEROPS database of proteolytic enzymes, their substrates and inhibitors in 2017 and a comparison with peptidases in the PANTHER database. Nucleic Acids Res 46:D624–D632. doi:10.1093/nar/gkx1134. 29145643 PMC5753285

[18] Lassy RAL, Miller CG. 2000. Peptidase E, a peptidase specific for N-terminal aspartic dipeptides, is a serine hydrolase. J Bacteriol 182:2536–2543. doi:10.1128/JB.182.9.2536-2543.2000. 10762256 PMC111318

[19] Richter R, Hejazi M, Kraft R, Ziegler K, Lockau W. 1999. Cyanophycinase, a peptidase degrading the cyanobacterial reserve material multi-l-arginyl-poly-l-aspartic acid (cyanophycin): molecular cloning of the gene of *Synechocystis* sp. PCC 6803, expression in *Escherichia coli*, and biochemical characterization of the purified enzyme. Eur J Biochem 263:163–169. doi:10.1046/j.1432-1327.1999.00479.x. 10429200

[20] Yadav P, Goyal VD, Chandravanshi K, Kumar A, Gokhale SM, Jamdar SN, Makde RD. 2019. Catalytic triad heterogeneity in S51 peptidase family: structural basis for functional variability. Proteins 87:679–692. doi:10.1002/prot.25693. 30968972

[21] Jumper J, Evans R, Pritzel A, Green T, Figurnov M, Ronneberger O, Tunyasuvunakool K, Bates R, Žídek A, Potapenko A, Bridgland A, Meyer C, Kohl SAA, Ballard AJ, Cowie A, Romera-Paredes B, Nikolov S, Jain R, Adler J, Back T, Petersen S, Reiman D, Clancy E, Zielinski M, Steinegger M, Pacholska M, Berghammer T, Bodenstein S, Silver D, Vinyals O, Senior AW, Kavukcuoglu K, Kohli P, Hassabis D. 2021. Highly accurate protein structure prediction with AlphaFold. Nature 596:583–589. doi:10.1038/s41586-021-03819-2. 34265844 PMC8371605

[22] Yadav P, Goyal VD, Gaur NK, Kumar A, Gokhale SM, Makde RD. 2018. Structure of Asp-bound peptidase E from *Salmonella enterica*: active site at dimer interface illuminates Asp recognition. FEBS Lett 592:3346–3354. doi:10.1002/1873-3468.13247. 30194851

[23] Ho SN, Hunt HD, Horton RM, Pullen JK, Pease LR. 1989. Site-directed mutagenesis by overlap extension using the polymerase chain reaction. Gene 77:51–59. doi:10.1016/0378-1119(89)90358-2. 2744487

[24] Kim J, Xiao H, Bonanno JB, Kalyanaraman C, Brown S, Tang X, Al-Obaidi NF, Patskovsky Y, Babbitt PC, Jacobson MP, Lee Y-S, Almo SC. 2013. Structure-guided discovery of the metabolite carboxy-SAM that modulates tRNA function. Nature 498:123–126. doi:10.1038/nature12180. 23676670 PMC3895326

[25] Nedev H, Nabarisoa H, Haertle T. 1993. A convenient method for synthesis of Fmoc-amino acid *p*-nitroanilides based on isobutyl chloroformate as condensation agent. Tetrahedron Lett 34:4201–4204. doi:10.1016/S0040-4039(00)60527-0.

[26] Peterson QP, Goode DR, West DC, Botham RC, Hergenrother PJ. 2010. Preparation of the caspase-3/7 substrate Ac-DEVD-pNA by solution-phase peptide synthesis. Nat Protoc 5:294–302. doi:10.1038/nprot.2009.223. 20134429 PMC2921128

[27] Marchler-Bauer A, Bo Y, Han L, He J, Lanczycki CJ, Lu S, Chitsaz F, Derbyshire MK, Geer RC, Gonzales NR, Gwadz M, Hurwitz DI, Lu F, Marchler GH, Song JS, Thanki N, Wang Z, Yamashita RA, Zhang D, Zheng C, Geer LY, Bryant SH. 2017. CDD/SPARCLE: functional classification of proteins via subfamily domain architectures. Nucleic Acids Res 45:D200–D203. doi:10.1093/nar/gkw1129. 27899674 PMC5210587

[28] Steinegger M, Söding J. 2017. MMseqs2 enables sensitive protein sequence searching for the analysis of massive data sets. Nat Biotechnol 35:1026–1028. doi:10.1038/nbt.3988. 29035372

[29] Edgar RC. 2004. MUSCLE: a multiple sequence alignment method with reduced time and space complexity. BMC Bioinformatics 5:113. doi:10.1186/1471-2105-5-113. 15318951 PMC517706

[30] Söding J. 2005. Protein homology detection by HMM-HMM comparison. Bioinformatics 21:951–960. doi:10.1093/bioinformatics/bti125. 15531603

[31] Price MN, Dehal PS, Arkin AP. 2010. FastTree 2—approximately maximum-likelihood trees for large alignments. PLoS One 5:e9490. doi:10.1371/journal.pone.0009490. 20224823 PMC2835736

