# Supplementary material for: S51 Family Peptidases Provide Resistance to Peptidyl-Nucleotide Antibiotic McC
Source: mBio. 2022 Apr 25;13(3):e00805-22. doi: 10.1128/mbio.00805-22 (PMC9239234; doi:10.1128/mbio.00805-22)
Supplement: FIG S1 [file mbio.00805-22-sf001.pdf]

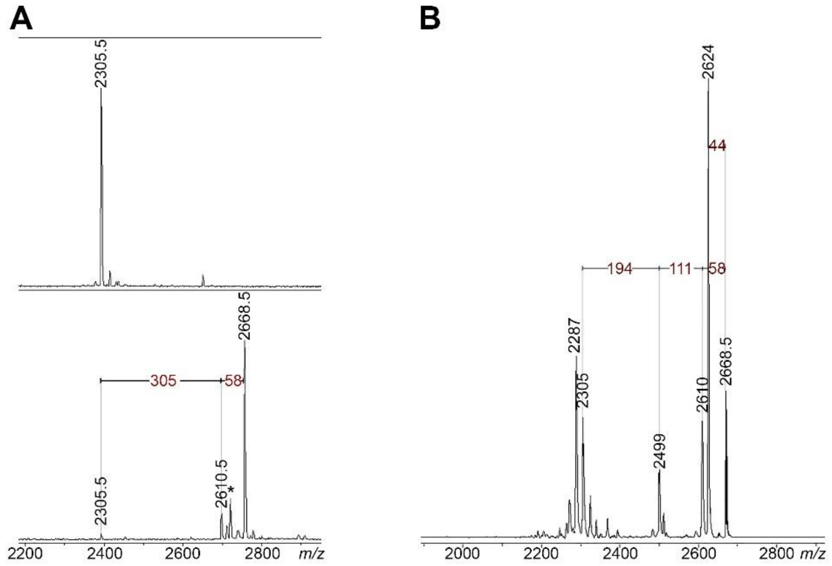

Figure S1. (A) MALDI MS spectra of synthetic MccA^{Nva} precursor peptide (upper panel) and the products of its *in vitro* modification by recombinant MccB^{Nva} (lower panel). The *in vitro* coupled nucleotidylation-carboxymethylation reaction was performed in the presence of a chemically synthesized cxSAM and equimolar mix of four NTPs (11). $[M+H]^+$ at *m/z* 2305.5 corresponds to unmodified MccA^{Nva}; mass-ions at *m/z* 2610.5 and at *m/z* 2668.5 match the MccA^{Nva}-cytidylate and carboxymethylated MccA^{Nva}-cytidylate, respectively. $[M+Na]^+$ at *m/z* 2632.5 marked with an asterisk corresponds to a sodium adduct of MccA^{Nva}-cytidylate. **(B)** MALDI TOF MS/MS spectrum of the products of MccA^{Nva} *in vitro* modification by MccB^{Nva} ($[M+H]^+$ ions at *m/z* 2668.5). Mass shifts for 44 and 58 Da correspond to removal of carboxyl and carboxymethyl groups, respectively. Mass differences in 111 and 194 Da match the loss of cytosine nucleobase and monophosphorylated ribose, correspondingly. $[M+H]^+$ ion at *m/z* 2287 matches dehydrated MccA peptide (MKIVLKLKRIVRGAGPIIVSN).
