# Supplementary material for: S51 Family Peptidases Provide Resistance to Peptidyl-Nucleotide Antibiotic McC
Source: mBio. 2022 Apr 25;13(3):e00805-22. doi: 10.1128/mbio.00805-22 (PMC9239234; doi:10.1128/mbio.00805-22)
Supplement: FIG S2 [file mbio.00805-22-sf002.pdf]

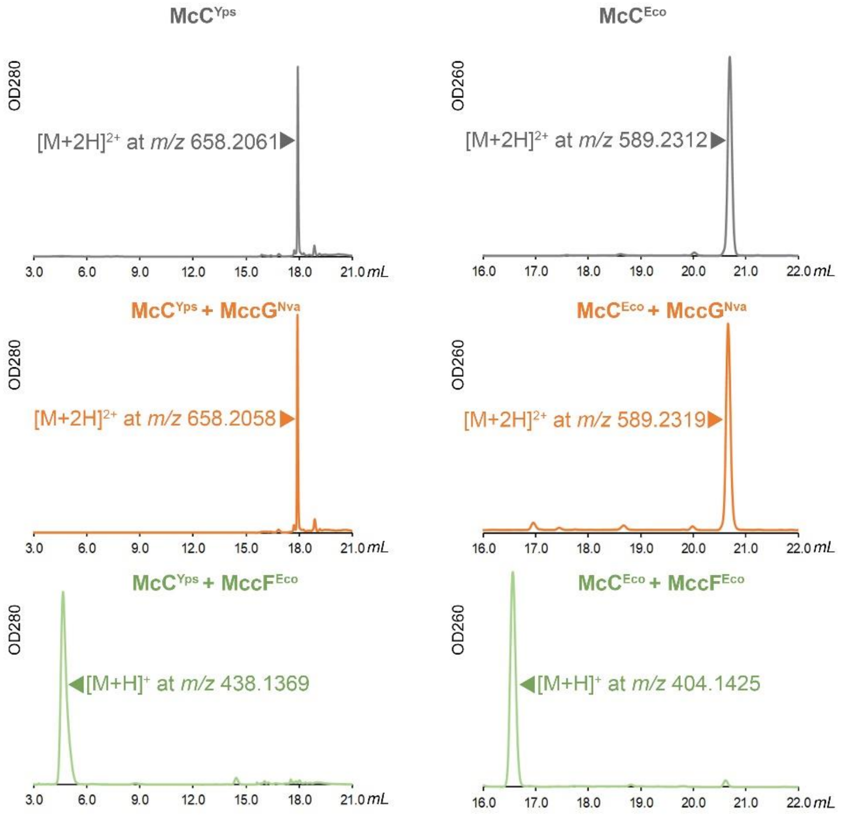

Figure S2. Intact McC-like compounds are not substrates for $MccG^{Nva}$. RP-HPLC elution profiles of McC^{Yps} ($[M+2H]^{2+}$ at *m/z* 658.2061 and $[M+3H]^{3+}$ at *m/z* 439.1395) and McC^{Eco} ($[M+2H]^{2+}$ at *m/z* 589.2312 and $[M+3H]^{3+}$ at *m/z* 393.1566) alone or after incubation with $MccG^{Nva}$ and $MccF^{Eco}$.
