# Supplementary material for: S51 Family Peptidases Provide Resistance to Peptidyl-Nucleotide Antibiotic McC
Source: mBio. 2022 Apr 25;13(3):e00805-22. doi: 10.1128/mbio.00805-22 (PMC9239234; doi:10.1128/mbio.00805-22)
Supplement: FIG S3 [file mbio.00805-22-sf003.pdf]

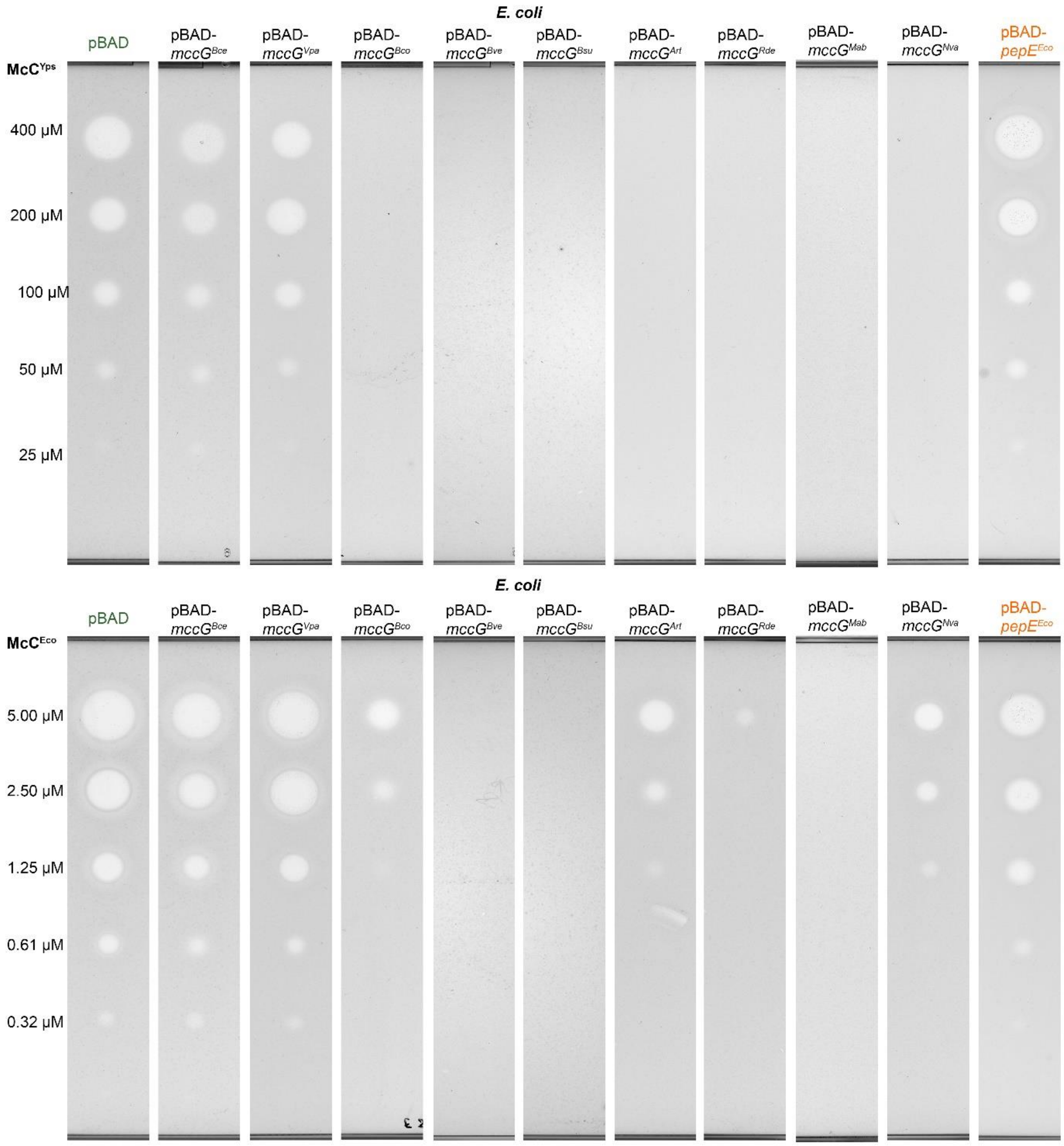

Figure S3. Susceptibility to McC^{Yps} and McC^{Eco} of *E. coli* harboring the plasmids with the indicated *mccG*^{Nva} homologs.
