# Supplementary material for: S51 Family Peptidases Provide Resistance to Peptidyl-Nucleotide Antibiotic McC
Source: mBio. 2022 Apr 25;13(3):e00805-22. doi: 10.1128/mbio.00805-22 (PMC9239234; doi:10.1128/mbio.00805-22)
Supplement: FIG S4 [file mbio.00805-22-sf004.pdf]

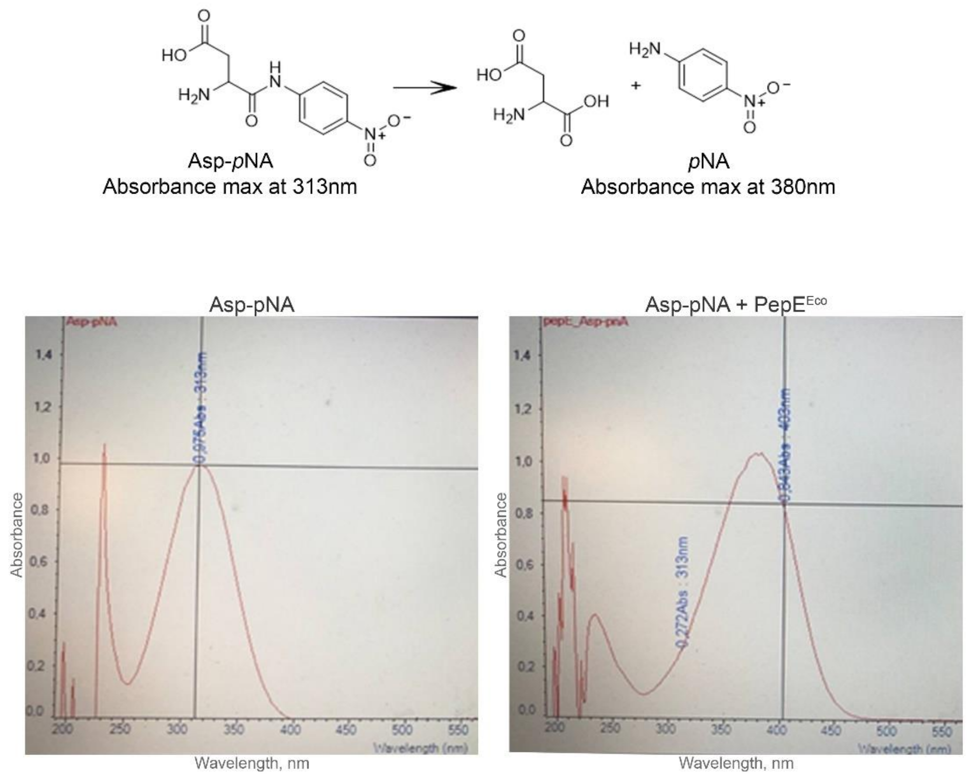

Figure S4. Recombinant PepE^{Eco} hydrolyzes synthetic Asp-*p*NA substrate. Absorbance spectra of Asp-pNA (left panel) and Asp-pNA incubated with PepE^{Eco} (right panel).
