# Supplementary material for: S51 Family Peptidases Provide Resistance to Peptidyl-Nucleotide Antibiotic McC
Source: mBio. 2022 Apr 25;13(3):e00805-22. doi: 10.1128/mbio.00805-22 (PMC9239234; doi:10.1128/mbio.00805-22)
Supplement: FIG S5 [file mbio.00805-22-sf005.pdf]

```
Cph          ------MPLSSQPAILIIGGAEDKVHGREILQTFW----SRSGGNDAIIGIIPSASREPL
PepEse       ------------MELLLLSN--STLPGKAWLEHALPLIANQLNGRRSAV-FIPFAGVTQT
MccG^Nva     MRRDESAGIDPVRRIILLGGGFSTDPDSLLDEYVL----SASAVDKPRVCFIPTASGDSR
                           ::::..  ..  .    :       .      . : :** *.

Cph          LIG--ERYQTIFSDMGVKELKVLDIRDRAQGDDSGYRLFVEQCTGIFMTGGDQLRLCGLL
PepEse       WDEYTDKTAEVLAPLGVNVTGI----HRVADPLA----AIEKAEIIIVGGGNTFQLLKES
MccG^Nva     --GYTDRFYSAFTRMNCTPSHLWLFHDRSADMAS----LVANQDILYVGGGSTANLLALW
                  :.    :: :. .   :     *  .  :     : :   : : **.  .*

Cph          ADTPLMDRIRQRVHNGEISLAGTSAGAAVMGHHMIAGGSSGEWPNRALVDMAV--GLGIV
PepEse       RERGLLAPMADRVKRGAL-YIGWSAGANL---ACPTIRTTNDMP---IVDPNGFDALDLF
MccG^Nva     RLHGLDRLIRDAYRRGVL-LCGISAGAACWFDACLTDSFGDLRP---LKD-----GLGLL
                 *   : :  ..* :   * ****        :    .  *   : *     .*.:.

Cph          PEIVVDQHFHNRNRMARLLSAISTHPELLGLGIDEDTCAMFERDGSVK--VIGQGTVSFV
PepEse       P-LQINPHFTN---------------------ALPEGHKGETREQRIRELLVVAPELTVI
MccG^Nva     E-GSFCPHFDAEPERDQLYGQAVSDGSLPGGWALQDGAAALFRNEELRDIVTRNGTSTII
                 .  **                          :      *:  :.  :      :.:

Cph          DA-RDMSYTNAA-----LVGANAPLSLHNLRLNILVHGEVYHQVKQRAFPRVT
PepEse       GL-PEGNWIQVSNGQAVLGGPNTTWVFKAGEEAVAL--EAGHRF---------
MccG^Nva     GMRRENGYTQIS--------------RKFSKAQILT--EPGGES---------
             .   : .: : :               :     :    *
```

Figure S5. A multiple sequence alignment of cyanophycinase from *Synechocystis* sp. (WP_010872518.1), the PepE peptidase from *S. enterica* (WP_000421792.1), and $MccG^{Nva}$ from *N. vaccinii* NBRC 15922 (WP_218027309.1) is presented. The alignment was built using MUSCLE with default parameters. Experimentally confirmed catalytic residues of Cph and PepE and are highlighted in red. Based on the sequence alignment, only two of these residues, a serine and a histidine are conserved in all three proteins.
