# Supplementary material for: S51 Family Peptidases Provide Resistance to Peptidyl-Nucleotide Antibiotic McC
Source: mBio. 2022 Apr 25;13(3):e00805-22. doi: 10.1128/mbio.00805-22 (PMC9239234; doi:10.1128/mbio.00805-22)
Supplement: FIG S6 [file mbio.00805-22-sf006.pdf]

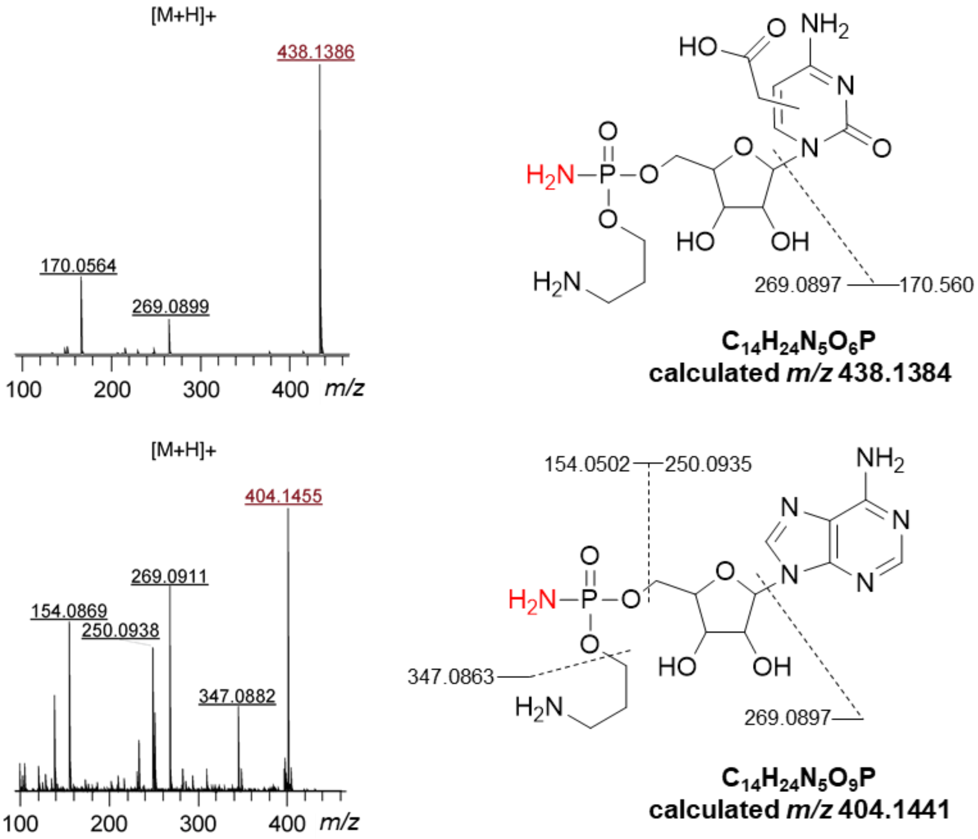

Figure S6. ESI-MS/MS fragmentation spectra of the products of $MccG^{Nva}$-mediated hydrolysis of the McC^{519} (upper panels) and McC^{553} (lower panels). Parent ions on mass-spectra are labeled with red color font.
