# Supplementary material for: S51 Family Peptidases Provide Resistance to Peptidyl-Nucleotide Antibiotic McC
Source: mBio. 2022 Apr 25;13(3):e00805-22. doi: 10.1128/mbio.00805-22 (PMC9239234; doi:10.1128/mbio.00805-22)
Supplement: FIG S7 [file mbio.00805-22-sf007.pdf]

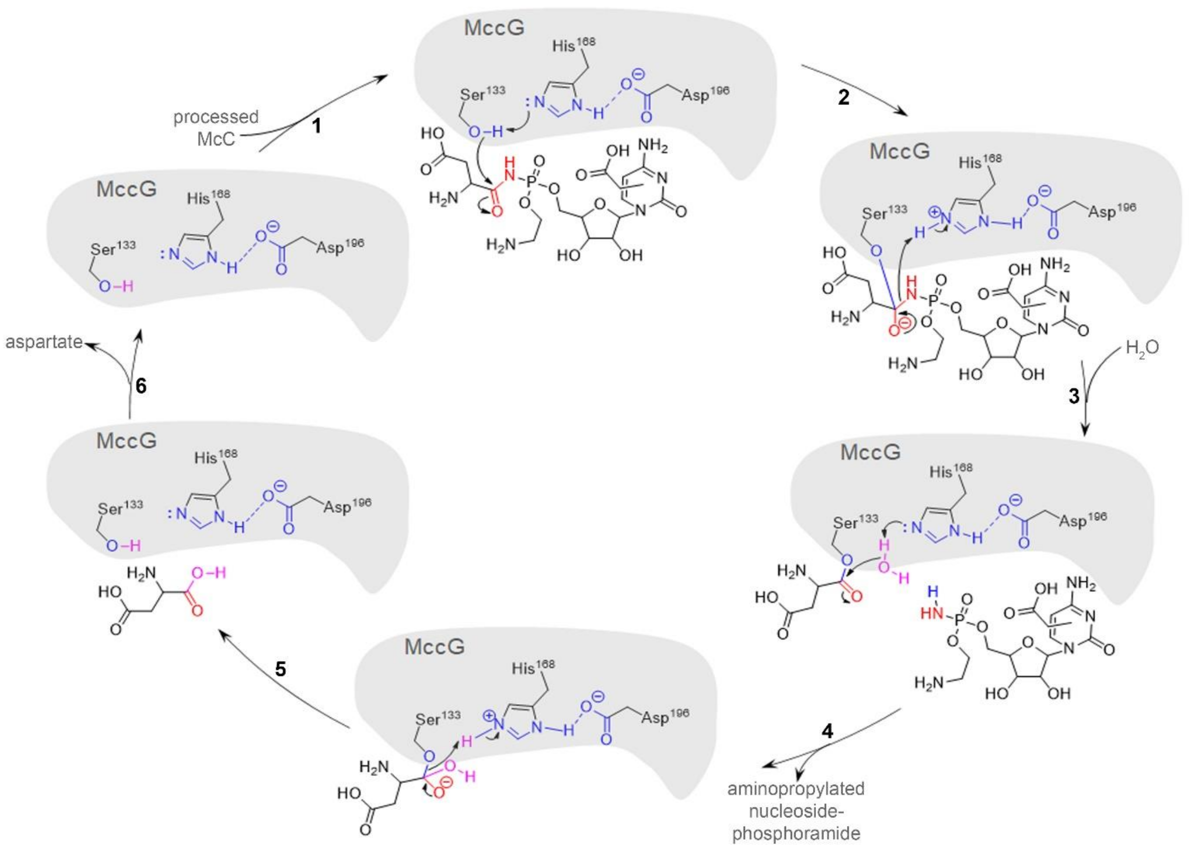

Figure S7. A proposed reaction mechanism of MccG. At the first step, Ser133 deprotonated with His168 attacks the carbonyl group of processed McC aspartate forming the first tetrahedral complex. The His168 complex with proton is stabilized via a hydrogen bond with the negatively charged Asp196. The His168 hydrogen is then taken by the nitrogen of the phosphamide bond of the substrate, releasing the nucleotide part. Next, the remaining covalently bound aspartate is attacked by a water molecule, which, similarly to Ser133 at the first step, is deprotonated by His168. The newly formed tetrahedral complex decomposes with the release of free aspartate and the catalytic triad is returned to its initial state.
