# Supplementary material for: S51 Family Peptidases Provide Resistance to Peptidyl-Nucleotide Antibiotic McC
Source: mBio. 2022 Apr 25;13(3):e00805-22. doi: 10.1128/mbio.00805-22 (PMC9239234; doi:10.1128/mbio.00805-22)
Supplement: TABLE S1 [file mbio.00805-22-st001.docx]

**Table S1. Primers used in the study**

| No | Primer ID | Sequence 5'-3' | Purpose |
| --- | --- | --- | --- |
| 1 | MccB_Nva_F_BamHI | TATTATGGATCCAAACGAATACTTGCAACTG | Cloning into pRSFDuet-1 |
| 2 | MccB_Nva_R_SacI | ATTATAGAGCTCTTAGCGGCCGAGCACGAACGTAAG |  |
| 3 | PepE_Eco_F_SalI | ATTTATGTCGACATGGAACTGCTTTTATTGAG | Cloning into pBAD_SalRBS |
| 4 | PepE_Eco_R_HindIII | TATATAAAGCTTAAAAACGGTGACCAGCTTCC |  |
| 5 | MccF_F_SalI | TATTATGTCGACATGATACAATCTCATCCACTAC |  |
| 6 | MccF_R_HindIII | TATTATAAGCTTATTTCTCGGTAGATAGATATTGTTCTG |  |
| 7 | MccG_F_SalI | ATTTATGTCGACATGCGTCGTGACGAGTCGGC |  |
| 8 | MccG_R_HindIII | ATATTATAAGCTTATGACTCCCCGCCCGGC |  |
| 9 | MccG_Art_F_SalI | ATTTATGTCGACATGGCAGCCCAACAGCCAAC |  |
| 10 | MccG_Art_R_HindIII | TATTAAAAGCTTAGCCAAGGAAACGGGCCTCC |  |
| 11 | MccG_Bsu_F_SalI | ATTATTGTCGACATGAAGCAGATTATTGCGATG |  |
| 12 | MccG_Bsu_R_HindIII | TATATTAAGCTTACCCTAAATATTTGACCGG |  |
| 13 | MccG_Bce_F_SalI | ATTATAGTCGACATGAAATTAGCTGTCATTGGTG |  |
| 14 | MccG_Bce_R_HindIII | TATATAAAGCTTATAAATAGCTTTTTAAAGTAATATC |  |
| 15 | MccG_Bco_F_SalI | ATTATTGTCGACATGAGGCAAATTATCGCAATG |  |
| 16 | MccG_Bco_R_HindIII | TATATTAAGCTTACATGTCATTATTATCATCTAAATATTTTA |  |
| 17 | MccG_Bve_F_SalI | ATTTATGTCGACATGACATTGAAGCAGATTATTGC |  |
| 18 | MccG_Bve_R_HindIII | ATTTATAAGCTTAAAATTCAGATAACGGTACGG |  |
| 19 | PepE_Eco_F_NdeI | ATTTATCATATGGAACTGCTTTTATTGAG | Cloning into pET22(b) |
| 20 | PepE_Eco_R_XhoI_wsc | TTATTTCTCGAGAAAACGGTGACCAGCTTCCAG |  |
| 21 | MccF_F_NdeI | TATTATCATATGATACAATCTCATCCACTAC |  |
| 22 | MccF_R_XhoI_wsc | ATTTTATCTCGAGTTTCTCGGTAGATAGATATTGTTCTG |  |
| 23 | MccG_F_NdeI | TATTTACATATGCGTCGTGACGAGTCGGC |  |
| 24 | MccG_R_SalI_wsc | ATATTATGTCGACTGACTCCCCGCCCGGCTC |  |
| 25 | MccG_S133A_F | GCTGCTGTGCGGCATCGCCGCCGGTGCCGCCTGCTG | Mutagenesis |
| 26 | MccG_S133A_R | CAGCAGGCGGCACCGGCGGCGATGCCGCACAGCAGC |  |
| 27 | MccG_H168A_F | GCAGTTTCTGCCCGGCGTTCGACGCCGAGCCCGAAC |  |
| 28 | MccG_H168A_R | GTTCGGGCTCGGCGTCGAACGCCGGGCAGAAACTGC |  |
| 29 | MccG_D196A_F | GAGGATGGGCCCTCCAGGCGGGCGCGGCCGCATTGTTTC |  |
| 30 | MccG_D196A_R | GAAACAATGCGGCCGCGCCCGCCTGGAGGGCCCATCCTC |  |
| 31 | MccG_D196E_F | GAGGATGGGCCCTCCAGGAAGGCGCGGCCGCATTGTTTC |  |
| 32 | MccG_D196E_R | GAAACAATGCGGCCGCGCCTTCCTGGAGGGCCCATCCTC |  |
